# Hypoxia inhibits ferritinophagy, increases mitochondrial ferritin, and protects from ferroptosis

**DOI:** 10.1016/j.redox.2020.101670

**Published:** 2020-08-03

**Authors:** Dominik C. Fuhrmann, Antonia Mondorf, Josefine Beifuß, Michaela Jung, Bernhard Brüne

**Affiliations:** a Institute of Biochemistry I, Faculty of Medicine, Goethe-University Frankfurt, Frankfurt, Germany; b Department of Internal Medicine 1, University Hospital Frankfurt, Germany; c Frankfurt Cancer Institute, Goethe-University Frankfurt, Frankfurt, Germany; d German Cancer Consortium (DKTK), Partner Site Frankfurt, Germany; e Branch for Translational Medicine and Pharmacology TMP of the Fraunhofer Institute for Molecular Biology and Applied Ecology IME, Frankfurt, Germany

**Keywords:** Macrophages, Iron, Hypoxia, FTMT, NCOA4, Ferritinophagy, Ferroptosis, miR-6862-5p, JNK

## Abstract

Cellular iron, at the physiological level, is essential to maintain several metabolic pathways, while an excess of free iron may cause oxidative damage and/or provoke cell death. Consequently, iron homeostasis has to be tightly controlled. Under hypoxia these regulatory mechanisms for human macrophages are not well understood. Hypoxic primary human macrophages reduced intracellular free iron and increased ferritin expression, including mitochondrial ferritin (FTMT), to store iron. In parallel, nuclear receptor coactivator 4 (NCOA4), a master regulator of ferritinophagy, decreased and was proven to directly regulate FTMT expression. Reduced NCOA4 expression resulted from a lower rate of hypoxic NCOA4 transcription combined with a micro RNA 6862-5p-dependent degradation of NCOA4 mRNA, the latter being regulated by c-jun N-terminal kinase (JNK). Pharmacological inhibition of JNK under hypoxia increased NCOA4 and prevented FTMT induction. FTMT and ferritin heavy chain (FTH) cooperated to protect macrophages from RSL-3-induced ferroptosis under hypoxia as this form of cell death is linked to iron metabolism. In contrast, in HT1080 fibrosarcome cells, which are sensitive to ferroptosis, NCOA4 and FTMT are not regulated. Our study helps to understand mechanisms of hypoxic FTMT regulation and to link ferritinophagy and macrophage sensitivity to ferroptosis.

## Highlights

• Hypoxia decreases NCOA4 transcription in primary human macrophages.
• NCOA4 mRNA is a target of miR-6862-5p.
• Lowering NCOA4 increases FTMT abundance under hypoxia.
• FTMT and FTH protect from ferroptosis.
• Tumor cells lack the hypoxic decrease of NCOA4 and fail to stabilize FTMT.

## Graphical abstract

**Figure:**
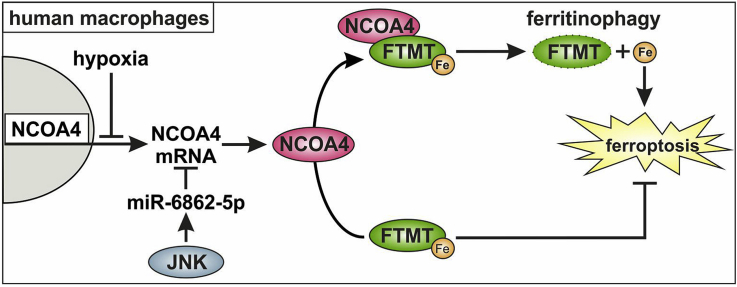

## 1 Introduction

Iron is critical for Fe–S cluster formation or heme synthesis and thus is essential to maintain the mitochondrial respiratory chain or the citric acid cycle [1,2]. Nevertheless, an excess of free iron may cause severe oxidative damage [3]. Thus, iron import, export, and storage have to be tightly controlled. Under hypoxia, which occurs in tumors or during inflammation, hypoxia inducible factors (HIF) are known to control iron regulatory proteins. HIF-regulated proteins are not only the transferrin receptor (TfR) and the divalent metal transporter 1 (DMT1), which are responsible for iron import, but also ferroportin (FPN), which is the only established iron exporter [[4], [5], [6], [7]]. Iron storage is equally important and may prevent cells from oxidative stress caused by an overload with redox active free iron. In this context three iron storage proteins are known: ferritin heavy chain (FTH), ferritin light chain (FTL), and mitochondrial ferritin (FTMT) [8,9]. FTH and FTL built protein complexes composed of 24 subunits. FTH as well as FTMT carry a ferroxidase activity, which allows storage of ferric hydroxides (Fe^3+^) instead of reactive ferrous iron (Fe^2+^) [10]. Iron loading of FTH is mediated by poly(rC)-binding protein 1 (PCBP1) [11]. FTH is regulated at transcriptional, translational, and protein level. Transcription of FTL and FTH under hypoxia or oxidative stress was associated with nuclear factor E2-related factor 2, which binds to antioxidant response elements upstream of the individual genes [12]. Translation of FTH and FTL is under the control of the iron regulatory protein (IRP) machinery. Under conditions of low iron, IRPs bind to an iron response element (IRE) in the 5’ UTR of the ferritin transcripts to block translation [13]. When sufficient iron becomes accessible, IRPs express aconitase activity and do not any longer bind to the IRE, which culminates in enhanced ferritin protein expression [14]. Iron release from ferritins is regulated by a process known as ferritinophagy. Ferritinophagy is mediated by nuclear receptor coactivator 4 (NCOA4), which directly binds FTH and transfers the complex to the autolysosome for degradation [[15], [16], [17], [18]]. NCOA4 has, in contrast to FTH, a low affinity to FTL indicating that ferritinophagy is predominantly regulated via the NCOA4-FTH system [19]. During ferritinophagy stored iron gets released and becomes available for biosynthetic pathways. NCOA4 abundance is controlled by the ubiquitin ligase HERC2 and degraded by the 26S-proteasome [20]. FTMT shows a high sequence homology to FTH, which implies a similar functionality. Nevertheless, mechanisms regulating FTMT are not completely understood. FTMT expression was associated with a shift of iron from the cytosol to mitochondria [21]. Moreover, its expression under hypoxia is HIF-dependent [22]. Macrophages often experience hypoxia, i.e. during inflammation or in the tumor microenvironment. Furthermore, they are known for their central role in iron metabolism, although regulation and functional consequences of macrophage ferritinophagy remains ill-defined [23]. Ferritin expression and ferritinophagy control divergent processes of iron storage vs. release and thus, are supposed to affect cell fate decision. Overexpression of FTMT protected SH-SY5Y neuroblastoma cells from ferroptosis, a form of cell death induced by iron, reactive oxygen species and lipid peroxidation [24,25].

Ferroptosis can be initiated by inhibiting phospholipid hydroperoxide glutathione peroxidase (GPX4) using RSL-3 or by interfering with cellular cystine uptake by Erastin [26,27]. Both inhibitors provoke lipid peroxide accumulation, disturb the GSH/GSSG system and in turn kill cells [28]. Generation of lipid peroxides is supposed to be lipoxygenase-dependent or to occurs as result of Fenton chemistry [29,30]. Fenton chemistry generates highly reactive hydroxyl radicals, which oxidize polyunsaturated fatty acids. GPX4 in turn reduces lipid peroxides and thus, protects from ferroptosis [31]. Besides GPX4, the ferroptosis suppressor protein 1 (FSP1, AIFM2) was shown to reduce CoQ_10_, which also blocks lipid peroxidation [32,33].

Here we show that an increase of FTMT in hypoxic human macrophages is linked to decreased NCOA4 expression. The NCOA4 decrease results from impaired transcriptional regulation under hypoxia and its mRNA degradation by micro RNA 6862-5p, which is controlled by c-jun N-terminal kinase (JNK). Functionally, an increased FTH and FTMT expression under hypoxia protects macrophages from ferroptosis.

## 2 Material and methods

### 2.1 Isolation of primary human macrophages

Primary human macrophages were isolated from Buffy coats using Leucosep tubes (Greiner bio-one, Frickenhausen, Germany) and Biocoll Separating Solution (Biochrom, Berlin, Germany). Cells were washed three times with PBS and were allowed to adhere to 6-well plates, 6 cm dishes, or 48-well plates (Cell+, Sarstedt, Nümbrecht, Germany) for 1 h at 37 °C. Non-adherent cells were removed and remaining monocytes were incubated for at least 7 days with RPMI 1640 medium containing 5% human serum and penicillin/streptomycin. Macrophages were used at a density of approximately 80%.

### 2.2 Cell culture HT1080

HT1080 fibrosarcoma cells were cultured in DMEM medium containing 10% fetal calf serum and penicillin/streptomycin. Cells were seeded 24 h before the experiment to ensure attachment.

### 2.3 siRNA transfection

Primary human macrophages were incubated in RPMI without human serum 16 h prior transfection. Cells were transfected with 50 nM siRNA against NCOA4 (ON-TARGETplus SMART pool, human NCOA4) or 25 nM miR-6862-5p antagomir (miRCURY, Qiagen, Hilden, Germany) using HiPerFect transfection reagent (Qiagen). HT1080 cells were transfected with 8 nM siRNA using jetPrime (Polyplus transfection, Illkirch, France) according to manufacturer's instructions.

### 2.4 Treatments

Cells were treated with SP600125, LY294002, SB203580, AKT VIII, Rapamycin (all from Sigma-Aldrich, Munich, Germany), RSL-3, or Liproxstatin-1 (both from Cayman Chemicals, Ann Arbor, USA) 1 h prior hypoxic incubations. Hypoxic incubations were performed in a SciTive Workstation (Baker Ruskinn, Leeds, UK) at 1% O_2_ and 5% CO_2_ for times indicated.

### 2.5 Western analysis

Cells were lysed in a buffer containing 4% SDS, 150 mM NaCl, and 100 mM Tris/HCl, pH 7.4, and sonicated. Protein content was determined by a protein assay kit (Bio-Rad, Munich, Germany) and 60 μg protein were loaded on a 10% SDS gel. Gels were blotted using a Trans Blot Turbo blotting system (Bio-Rad). Before blocking membranes were stained using the Revert™ 700 Total Protein Stain kit (Licor, Lincoln, USA) according to manufacturer's advices. Afterwards, membranes were blocked in 5% milk in TBS-T for NCOA4 (A302-272A, Biomol, Hamburg, Germany), FTH (ab65080, abcam, Berlin, Germany), FTL (ab80585, abcam), or 5% bovine serum albumin in TBS-T for FTMT (ab66111, abcam). Fluorescence signal was detected on an Odyssey scanner (Licor) and quantified with Image Studio Digits 5.0 (Licor). For each lane the lane normalization factor (LNF) was calculated (intensity of a complete lane divided by the intensity of the lane with the maximal intensity) and used for normalization of the signal. For publication, the prominent band at 55 kDa was used. Complete total protein stains are collectively shown in Fig. S1.

### 2.6 Real time PCR

RNA was isolated using peqGold (Peqlab, Erlangen, Germany) and measured using a Nanodrop ND-1000 spectrophotometer (Peqlab). Reverse transcription was performed with the Maxima First Strand cDNA Synthesis Kit for RT-PCR (Thermo Fisher Scientific, Waltham, USA). For Reverse transcription of micro RNA the MystiCq microRNA cDNA Synthesis Mix (Sigma-Aldrich) was used. RNA expression of DMT1, TfR, CP, FPN, FTH, FTMT, FTL, HERC2, PCBP1, NCOA4, and miR-6862-5p was analyzed using PowerUp SYBR Green Master Mix (Applied Biosystems, Thermo Fisher Scientific) on a QuantStudio 3 PCR Detection System (Applied Biosystems, Thermo Fisher Scientific) and normalized to TBP. Primers are listed in Table 1. SNORD44 primers were purchased from Sigma.

**Table 1:** List of primers.

	Forward (5′-3′)	Reverse (5′-3′)
TBP	GCATCACTGTTTCTTGGCGT	CGCTGGAACTCGTCTCACTA
DMT1	AGCCACTCAGGTATCCACCAT	CCAGGGGACTGTGAAAGAGAG
TfR	GAGCGTCGGGATATCGGGT	CAGGATGAAGGGAGGACACG
CP	CTTTCCTGCTACCCTGTTTGATGC	CTTGCAAACCGGCTTTCAGA
FPN	TGAGCCTCCCAAACCGCTTCCATA	GGGCAAAAAGACTACAACGACGACTT
FTH	TGTGGCGGAGCTGCTGGGTAA	CGAGAGGTGGATACGGCTGCT
FTL	AGCCTTCTTTGTGCGGTCGGGTAA	ACGCCTTCCAGAGCCACATCAT
FTMT	CATGCCATGGAGTGTGCTCT	AATCGCACAAATGGGGGTCA
HERC2	TGCTTCCTACTTAGGCGTGC	TGGCAAGAGAAGGGCGATTT
PCBP1	TCGGCTTCTTATGCACGGAA	GGCCGGTCAGAGTGATGATT
NCOA4	TCAAGATGTAACCGTTGGGAA	AGCAGAAAGGCTGCTCAACT
miR-6862-5p	CGGGCATGCTGGGAGAGACTTT	MystiCq Universal Primer

### 2.7 Atomic absorbtion sprectroscopy

Cellular iron or iron in the supernatant was determined by graphite furnace atomic-absorption-spectrometry. Samples were measured as triplicate with a PinAAcle™ 900 T atomic-absorption-spectrometer (PerkinElmer, Rodgau, Germany). A wavelength of 248.33 nm and a slit width of 0.2 mm were used as spectrometer parameters. A hollow cathode iron lamp (30 mA maximum operating current) was run at 100% maximum current. The calibration solutions (10 μg/l to 90 μg/l) were prepared by adequate dilution of iron standard for AAS (Sigma-Aldrich) stock solution. A pyrolysis temperature of 1400 °C and an atomization temperature of 2100 °C were used. The iron amount was quantified relative to the protein content.

### 2.8 Chromatin immunoprecipitation

For ChIP analysis 15 cm dishes with primary human macrophages were harvested after 4 h (HIF-ChIPs) or 8 h and 48 h (Pol II-ChIPs) under hypoxia. ChIP experiments were carried out as previously described [34]. Briefly, cross-linking was performed in hypoxia using 1% formaldehyde for 10 min at 37 °C and quenched by 0.125 M glycine. Cells were then collected after 2 washing steps in PBS and centrifuged at 500×*g* for 5 min. Pellets were frozen at -80 °C. Cells were resuspended in 1.5 ml L1A buffer (10 mM HEPES/KOH, pH 7.9, 85 mM KCl, 1 mM EDTA, pH 8.0, 1 mM PMSF, 2 mM DTT, and protease inhibitor cocktail from Roche, Mannheim, Germany) and lysed in 250 μl of L1B buffer (L1A + 1% Nonidet P-40) for 15 min on a roller mixer at 4 °C. Cross-linked chromatin was sheared to an average DNA fragment size around 200–500 bp using a Branson Sonifier 250 (Dietzenbach-Steinberg, Germany). DNA quality was estimated by electrophoresis. After centrifugation, 5% of the supernatant was used as an input. After preclearing with Sepharose CL-4B beads for 2 h, equal amounts of chromatin were immunoprecipitated overnight with 10 μl anti-Pol II (Cell Signaling, Frankfurt Germany), anti-H3 (Merck Millipore, Darmstadt, Germany), or anti-rabbit IgG (Merck Millipore). Immune complexes were recovered by 2 h incubations with protein A-Sepharose CL-4 beads at 4 °C. After reverse cross-linking, DNA was purified using QIAquick PCR purification kits (Qiagen) according to instructions provided. Enrichment of specific DNA fragments in the immunoprecipitated material was determined by qPCR on a QuantStudio 3 PCR Detection System (Applied Biosystems, Thermo Fisher Scientific).

### 2.9 Vitality assay

To analyze the viability of the cells, cells were stained with CellTiter Blue (Promega, Walldorf, Germany) and incubated for 1 h under normoxic cell culture conditions. Afterwards, fluorescence was measured on an Infinite 200 pro plate reader (Tecan, Männedorf, Switzerland).

### 2.10 Statistics

Statistics were performed with GraphPad Prism 8.2.1. Data are expressed as mean values ± SEM. Statistically significant differences were calculated after analysis of variance (ANOVA) and Bonferroni's test or Students t-test; p < 0.05 was considered significant.

## 3 Results and discussion

### 3.1 Iron handling in macrophages under hypoxia

**Fig. 1:**
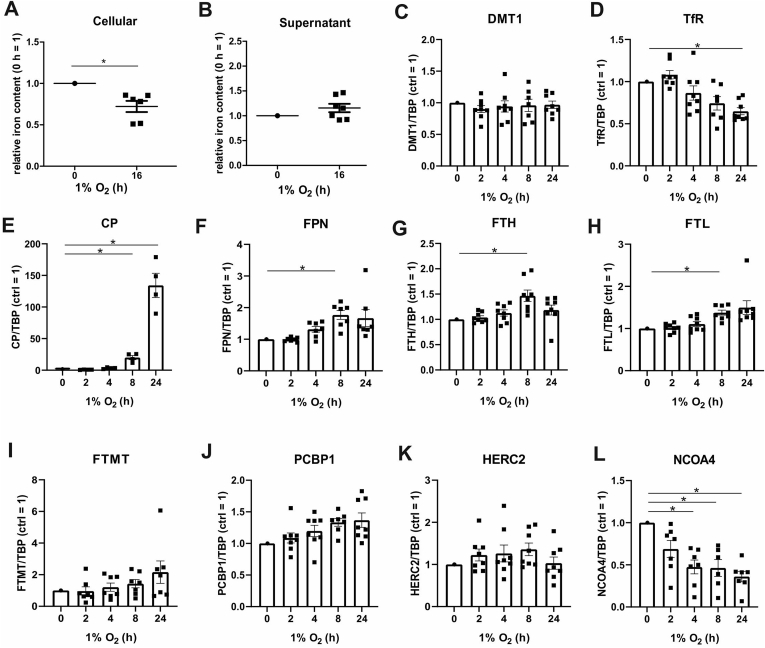
Regulation of iron and associated genes under hypoxia. A. Primary human macrophages were incubated under hypoxia (1% O_2_, 16 h). Cellular iron was determined by atomic absorption spectroscopy. Data were normalized to the normoxic control (n = 6–7). B. Primary human macrophages were incubated for 16 h under hypoxia (1% O_2_) and iron in the cell supernatants was analyzed by atomic absorption spectroscopy (n = 6–7). Data were normalized to the normoxic control. C-L. Human macrophages were incubated for indicated times under hypoxia (1% O_2_), RNA of divalent metal transporter 1 (DMT1), transferrin receptor (TfR), ceruloplasmin (CP), ferroportin (FPN), ferritin heavy chain (FTH), ferritin light chain (FTL), mitochondrial ferritin (FTMT), poly(rC)-binding protein 1 (PCBP1), ubiquitin ligase E3 ubiquitin-protein ligase HERC2 (HERC2), and nuclear receptor coactivator 4 (NCOA4) was analyzed by qPCR, and normalized to TATA box binding protein (TBP). Date were normalized to the normoxic control (n = 7–8). Data are mean values with SEM. Students t-test values p < 0.05 were considered as significant.

Under physiological, homeostatic conditions there is a demand for iron to serve several metabolic pathways. On the other side iron can also cause formation of reactive oxygen species and lipid peroxidation under conditions linked to various pathologies such as ischemia/reperfusion, kidney injury, or cancer. To explore effects of hypoxia on iron homeostasis, we used human macrophages to determine iron in cells and the cell supernatants by atomic absorption spectroscopy (Fig. 1A and B). After 16 h of hypoxia, intracellular iron decreased compared to normoxic controls, while iron in the supernatant slightly increased. Apparently, macrophages acquire an iron export phenotype under hypoxic conditions. This is supported by previous studies, which identified the iron exporter ferroportin (FPN) as a HIF-2 target gene [4]. To address the expression of iron regulatory genes under hypoxia we followed their mRNA levels in primary human macrophages incubated for 2–24 h under hypoxia compared to a normoxic control. Divalent metal transporter 1 (DMT1) and the transferrin receptor (TfR) are involved in iron import. While DMT1 mRNA expression remained unchanged, TfR mRNA decreased from 4 h onwards, which reached significance at 24 h hypoxia (Fig. 1C and D). Genes pointing to iron export like ceruloplasmin (CP) and FPN significantly increased, starting at 8 h (Fig. 1E and F). Alterations of these iron import/export associated genes support our notion of reduced intracellular iron under hypoxia. To complete our picture, we also analyzed genes known to affect iron storage. mRNA expression of ferritin heavy chain (FTH) and light chain (FTL) moderately increased and reached significance at 8 h hypoxia (Fig. 1G and H). In contrast, the mRNA of mitochondrial ferritin (FTMT) was not regulated under hypoxia, which makes a direct regulation of FTMT by HIF in human macrophages unlikely, although a hypoxia-responsive element in FTMT was reported by the group of Wu for mouse brain tissue (Fig. 1I) [22]. Similarly, the mRNA content of PCBP1, which loads ferritin with iron, and the E3 ubiquitin-protein ligase HERC2 (HERC2), regulating ubiquitin-dependent nuclear NCOA4 turnover during excess iron, were not changed under hypoxia (Fig. 1J and K). mRNA expression of NCOA4, which is a selective cargo receptor known to guide ferritin to the autolysosome, rapidly decreased after 2 h of hypoxia and reached significance after 4 h (Fig. 1L) [18,19]. The decrease of NCOA4 mRNA may suggest a link between ferritinophagy and hypoxia.

### 3.2 Decreased ferritinophagy under hypoxia

**Fig. 2:**
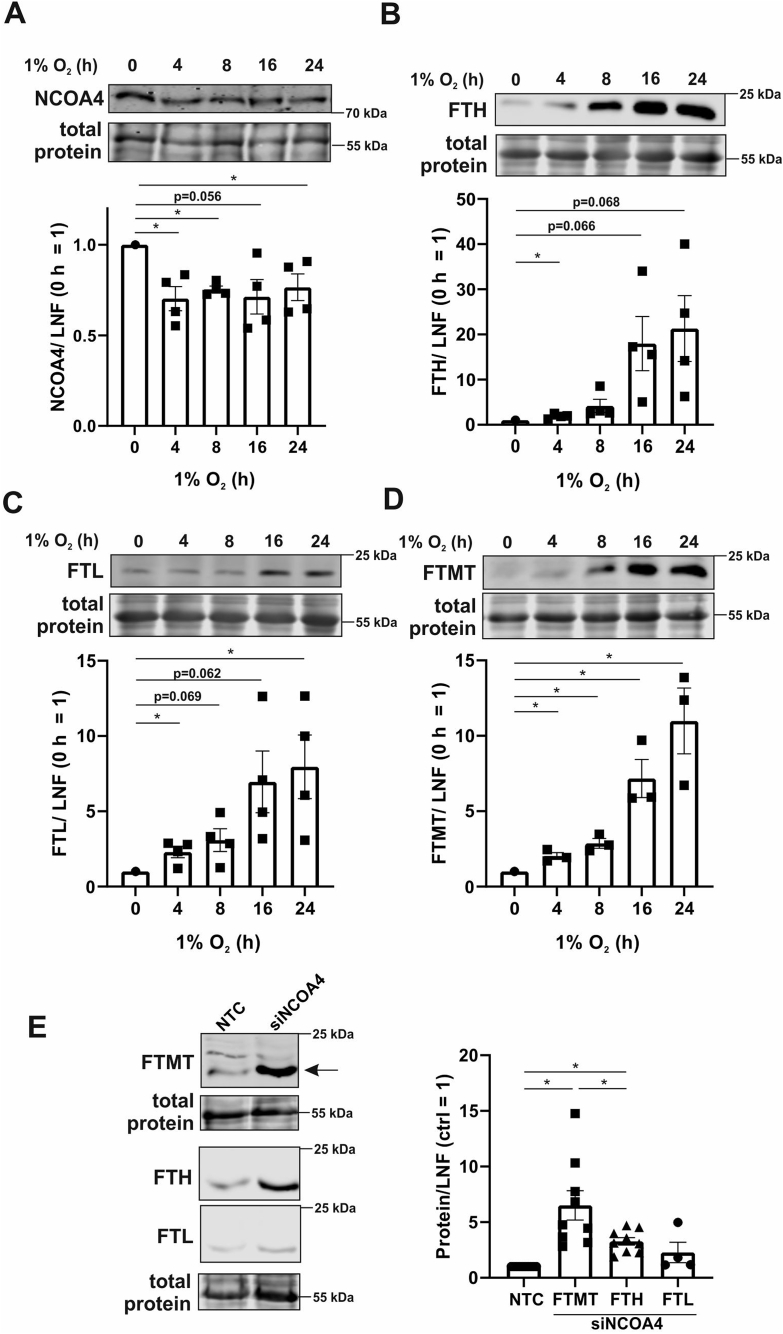
Protein expression of ferritinophagy related proteins. A-D. Primary human macrophages were incubated for indicated times under hypoxia (1% O_2_), total protein was stained, and Western analysis was performed for nuclear receptor coactivator 4 (NCOA4), ferritin heavy chain (FTH), ferritin light chain (FTL), and mitochondrial ferritin (FTMT). For quantification, the lane normalization factor (LNF) was calculated and the normoxic control was set to 1 (n = 3–4). E. Macrophages were transfected with a siRNA against NCOA4 (siNCOA4) or an untargeted control (NTC). Total protein was stained and Western analysis for FTMT, FTH, and FTL was performed. For quantification, the intensity was normalized to LNF and NTC was set to 1 (n = 4–9). Complete total protein stains are collectively shown in Fig. S1. All data are depicted with SEM. Students t-test values p < 0.05 were considered as significant.

We then followed expression of NCOA4 by Western analysis to explore whether ferritinophagy is limiting under hypoxia (Fig. 2A). Confirming mRNA data, NCOA4 protein significantly decreased at 4 h hypoxia. NCOA4 is identified as a cargo receptor, activated by autophagy-related proteins (ATG), which guides FTH and FTL to the autophagosome [15]. The group of Gryzik recently demonstrated that NCOA4 predominantly binds FTH by determining the ferritin binding domain of NCOA4 and analyzing its binding capacities to FTH and FTL [19]. In ATG-deficient cells NCOA4 accumulated, co-localized with autolysosomes, and was found to bind FTH [17]. In support of these considerations, NCOA4 and PCBP1 are crucial for iron liberation from storage proteins and thus, are needed for heme biosynthesis in erythrocytes [16]. Since NCOA4 is involved in degrading ferritins, a lower expression of NCOA4 should enhance ferritin abundance under hypoxia. Therefore, we analyzed FTH, FTL, and FTMT at protein level (Fig. 2B–D). Indeed, all ferritin isoforms significantly increased after 4 h hypoxia, which became more pronounced after 16–24 h. Of note, protein expression of FTH and FTL was seen prior to respective mRNA alterations, which makes a transcriptional or translational effect of ferritin regulation under these conditions less likely. Moreover, FTMT mRNA was not regulated at all, with only its protein expression changing. To validate the assumption, that NCOA4 regulates FTH, but also FTMT expression, cells were transfected with a siRNA against NCOA4 (siNCOA4) or a non-targeting control siRNA (NCT) (Fig. S3A). Knocking down NCOA4 strongly enhanced FTH and FTMT protein expression (Fig. 2E), while FTL was not significantly induced under these conditions. This fits the observations of Gryzik [19] and justified to excluded FTL from further analyses. The observation that a decrease of NCOA4 either by genetic manipulation or under hypoxia provokes a FTMT increase had not been reported before. Besides confirming FTH as an established NCOA4 target, we now provide evidence that also FTMT is under the control of NCOA4. Enhanced iron export coupled to a lower import in association with ferritin stabilization may indicate that macrophages use this mechanism to reduce the intracellular labile iron pool under hypoxia in order to reduce redox stress.

### 3.3 JNK-dependent regulation of NCOA4

**Fig. 3:**
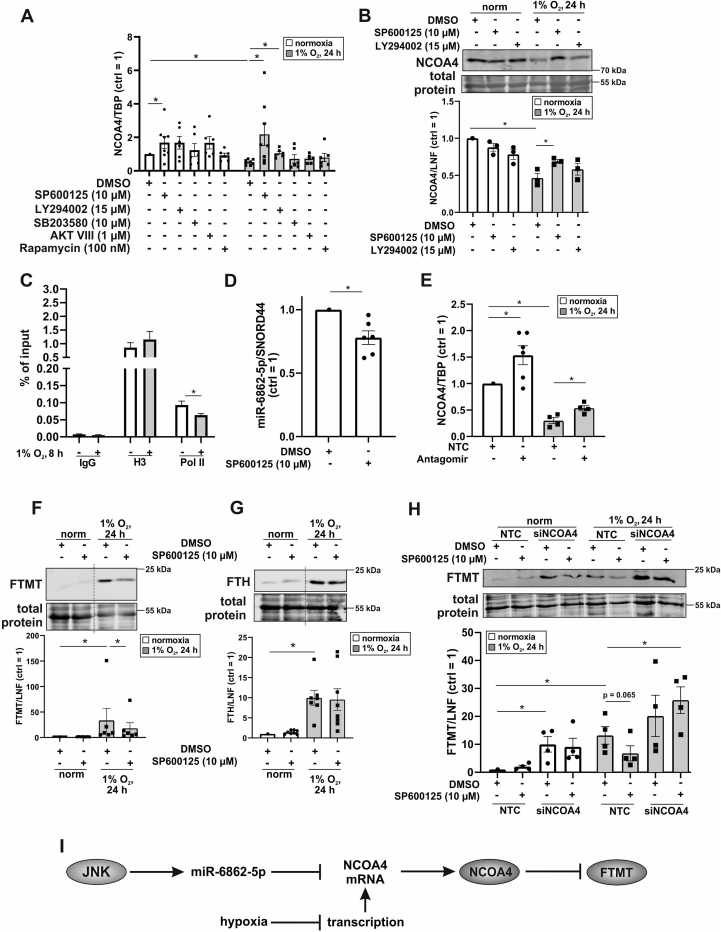
NCOA4 mediates degradation of FTMT under hypoxia. A. Primary human macrophages were treated with SP600125 (10 μM), LY294002 (15 μM), SB203580 (10 μM), AKT VIII (1 μM), or Rapamycin (100 nM) 1 h prior to hypoxic incubation (1% O_2_, 24 h). The mRNA of nuclear receptor coactivator 4 (NCOA4) was analyzed by qPCR and normalized to TBP. The DMSO control was set to 1 (n = 6–8). B. Primary human macrophages were treated with DMSO, SP600125, or LY294002 1 h prior to hypoxic incubation (1% O_2_, 24 h). Total protein was stained and NCOA4 was analyzed by Western blotting. For quantification, the lane normalization factor (LNF) was calculated and the normoxic DMSO control was set to 1 (n = 3). C. Cells were incubated under hypoxia (1% O_2_, 8 h) and chromatin immunoprecipitation was performed for immune globulin G (IgG), histon H3 (H3), and polymerase II (Pol II). The precipitate was probed for NCOA4 and data were expressed as percent of input (n = 3). D. Cells were incubated with SP600125 for 24 h and micro RNA (miR) 6862-5p was analyzed by qPCR. Data were normalized to SNORD44 and DMSO control was set to 1 (n = 6). E. Macrophages were transfected with an antagomir for miR-6862-5p (25 nM) and NCOA4 mRNA was analyzed (n = 4). F-G. Primary human macrophages were treated with SP600125 1 h prior to hypoxic incubation (1% O_2_, 24 h). Total protein was stained and mitochondrial ferritin (FTMT) and ferritin heavy chain (FTH) were analyzed by Western blotting. For quantification, protein expression was normalized to LNF and the normoxic control was set to 1 (n = 6–8). For reasons of clarity the blot was cut at the dashed line. H. Cells were transfected with an siRNA against NCOA4 for 24 h and treated with SP600125 1 h prior to hypoxic incubations (1% O_2_, 24 h). Western analysis was performed for FTMT and protein expression was normalized to LNF. DMSO control was set to 1 (n = 4). Complete total protein stains are collected in Fig. S1. I. Schematic outline of results. All data are depicted with SEM. Students t-test or ANOVA values p < 0.05 were considered as significant.

To substantiate the idea that a decrease in ferritinophagy under hypoxia accounts for the increase in ferritins, we explored NCOA4 regulation. For this propose, we inhibited c-Jun N-terminal kinase (JNK) using SP600125, phosphoinositide 3-kinase with LY294002, p38 mitogen-activated protein kinase with SB203580, protein kinase B employing AKT VIII, and mammalian target of rapamycin with Rapamycin and measured NCOA4 mRNA expression under normoxia and hypoxia (Fig. 3A). The potency of the individual inhibitors was confirmed in a separate experiment (Fig. S2A). As anticipated, NCOA4 mRNA was lower under hypoxia with the notion that only SP600125 and LY294002 rescued this decrease. Inhibition of JNK by SP600125 also increased NCOA4 mRNA under normoxia, arguing for the contribution of JNK to negatively regulate NCOA4. These findings were then validated at protein level (Fig. 3B). Downregulation of NCOA4 under hypoxia was effectively antagonized when blocking JNK with SP600125. LY264002 produced only minor effects in accordance with a similarly lower mRNA enhancement. Under normoxia no apparent changes in protein abundance with these inhibitors occurred. Since SP600125 increased NCOA4 mRNA in hypoxic and normoxic samples this pathway alone cannot explain the NCOA4 mRNA decrease under hypoxia. Therefore, we considered transcriptional regulation of NCAO4 mRNA under hypoxia. We performed chromatin immunoprecipitation (ChIP) analyses by precipitating polymerase II (Pol II) and probing the IP for NCOA4. Immune globulin G (IgG) served as a negative control and reveled no difference comparing normoxia to hypoxia. As a positive control, Histone H3 (H3) was immunoprecipitated, providing an enhanced but similar signal in hypoxic and normoxic samples. However, the IP showed a significantly decreased abundance of Pol II bound to the NCOA4 gene under hypoxia, suggesting that transcription of NCOA4 is hypoxia sensitive. Conclusively, blocking *de novo* NCOA4 mRNA synthesis in combination with its JNK-dependent reduction may account for the rapid decrease of NCOA4 levels under hypoxia. Mechanistically, the question how JNK decreases NCOA4 mRNA remains an open issue.

### 3.4 NCOA4 mRNA is a target of miR-6862-5p

JNK is known to increase the transcription of several target genes [35,36], with the possibility that JNK also enhances the expression of micro RNAs (miR). An in silico prediction of miRs that might target NCOA4 pointed to miR-6862-5p as a candidate. As this miR was not studied before no defined targets exist. If JNK increases miR-6862-5p one would predict this response to be sensitive to inhibition with SP600125. Indeed, blocking JNK significantly reduced the amount of miR-682-5p (Fig. 3D). In a next step, by transfecting macrophages with an antagomir towards miR-6862-5p or a control miR (NTC), we directly linked this miRNA to NCOA4 expression. Antagonizing miR-6862-5p significantly increased NCOA4 mRNA under normoxia and hypoxia. (Fig. 3E). To estimate the efficiency of the antagomir, expression of miR-6862-5p and its predicted target thrombomodulin (THBD) were measured (Figs. S2B and C). While the antagomir significantly reduced miR-6862-5p expression it increased mRNA content of THBD under normoxia. Since THBD is responsive to hypoxia, the antagomir was not effective under conditions of low oxygen. These data identified JNK as a regulator of miR-6862-5p and validated NCOA4 as well as THBD as novel targets of miR-6862-5p in human macrophages.

### 3.5 Stabilization of NCOA4 reduces FTMT but not FTH expression under hypoxia

Taking into account that NCOA4 degrades FTH by autophagy, we hypothesized that also FTMT is regulated by NCOA4 under hypoxia. Therefore, we analyzed the expression of FTMT and FTH under hypoxia in SP600125-treated macrophages (Fig. 3F and G). FTMT or FTH expression under normoxia was not affected by these inhibitors. The increase of FTMT under hypoxia was reversed, when blocking JNK. FTH increased under hypoxia as well but SP600125 was unable to interfere. Conclusively, only FTMT, but not FTH is regulated by NCOA4 under hypoxia. We went on and used a siRNA approach to knockdown NCOA4 in combination with blocking JNK activity. Whereas SP600125 had no effect on FTMT expression under normoxic conditions, the knockdown of NCOA4 increased FTMT protein expression to levels seen under hypoxia (Fig. 3H). Under hypoxia, SP600125 decreased NCOA4 protein amount, while the combination of knocking down NCOA4 and blocking JNK allowed recovering FTMT abundance under hypoxia. As a result, miR-6862-5p targets NCOA4 mRNA, an effect mediated by JNK. Since NCOA4 mRNA increased by SP600125 under normoxia and hypoxia, we consider a basal activity of miR-6862-5p, which is hypoxia independent. Thus, the rapid decline of NCOA4 mRNA under hypoxia appears as a combination of the continuous mRNA degradation by miR-6862-5p and the hypoxic attenuated transcription. As a consequence of reduced NCOA4 under hypoxia ferritinophagy is decreased, with a concomitant increase of FTMT (Fig. 3I). Since hypoxic induction of FTH was unrelated to NCOA4 (Fig. 2, Fig. 3G) and less cellular iron was measured in macrophages (Fig. 1A), we suspect that it is elevated by an IRP-dependent increase in translation [37,38]. We conclude that inhibition of ferritinophagy is an essential element to increase FTMT expression under hypoxia. In fibroblasts, Hou et al. connected autophagy to ferroptosis by showing that Erastin-mediated ferroptosis was diminished by a knockdown of ATG5 and 7 [39]. Additionally, NCOA4 expression was inhibited, which reduced degradation of ferritin and suppressed ferroptosis. The ferroptosis-enhancing role of autophagy was strengthened by the group of Park, who attenuated ferroptosis in murine lung fibroblasts by inhibiting autophagy and thus, ferritin degradation [40]. It was further proven that a knockdown of FTH but not FTL led to a growth deficiency in the larval wing discs of *Drosophila*, which was triggered by the enhanced production of reactive oxygen species and subsequent ferroptosis [41]. Interestingly, *Drosophila*, which overexpress FTMT were protected from ferroptosis induced by an Erastin-rich diet [25]. Conclusively, in *Drosophila* FTMT and FTH protect from ferroptosis. As our data showed enhanced expression of FTMT and FTH under hypoxia, we asked, whether macrophages cultured under hypoxia develop a higher degree of resistance to ferroptosis compared to cells cultured under normoxia.

### 3.6 RSL-3 increases FTMT and FTH

**Fig. 4:**
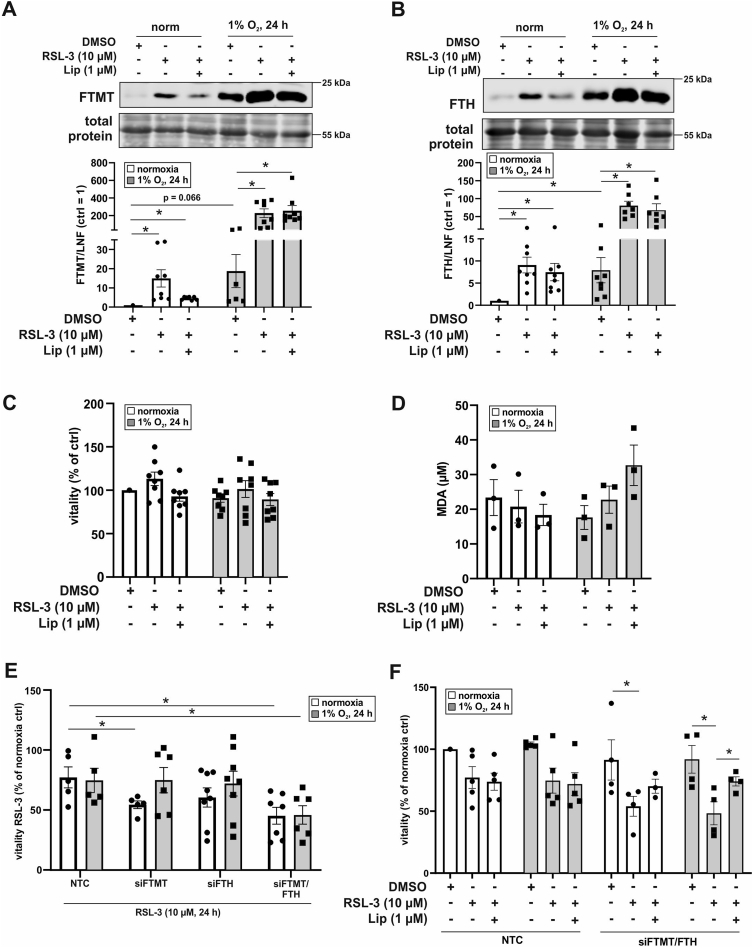
Ferroptosis in human macrophages. A-B. Cells were treated with RSL-3 (10 μM) and Liproxstatin-1 (Lip, 1 μM) and incubated under hypoxia (1% O_2_, 24 h). Expression of mitochondrial ferritin (FTMT) and ferritin heavy chain (FTH) were analyzed by Western blotting and normalized to the lane normalization factor (LNF). The DMSO control was set to 1 (n = 7–8). C. Macrophages were treated as described for A-B and vitality was analyzed after 1 h incubation with Cell titer blue (n = 8). The DMSO control was set to 100%. D. Cells were treated as mentioned in A and malondialdehyde (MDA) was measured (n = 3). E. Human macrophages were transfected with a siRNA against FTMT and/or FTH or an untargeted control, treated with RSL-3, and incubated under normoxia/hypoxia (1% O_2_, 24 h). Vitality of the cells was analyzed by CellTiter-Blue staining. The normoxic DMSO control was set to 100% (n = 5–8). F. Cells were transfected with siRNAs against FTMT and FTH or a nontargeting control and treated as mentioned in A. Vitality of the cells was analyzed by CellTiter-Blue staining. The normoxic DMSO control was set to 100% (n = 4–5). Complete total protein stains are collected in Fig. S1. All data are depicted with SEM. Students t-test or ANOVA values p < 0.05 were considered as significant.

We started to determine expression of FTMT and FTH after treating macrophages with the GPX4 inhibitor and ferroptosis inducer RSL-3 ((1S,3R)-2-(2-chloroacetyl)-2,3,4,9-tetrahydro-1-[4-(methoxycarbonyl)phenyl]-1H-pyrido[3,4-b]indole-3-carboxylic acid, methyl ester) (Fig. 4A and B). RSL-3 was used in combination with Liproxstatin-1 (Lip), known to protect from lipid peroxidation and consequently inhibits ferroptosis. Under normoxia RSL-3 induced FTMT as well as FTH, which was largely reverted by co-incubating RSL-3 with Liproxstatin-1. Hypoxia enhanced the protein amount of FTMT and FTH and this increase was further raised by RSL-3 but became insensitive to Liproxstatin-1. Taking the remarkable increase of ferritins and their assumed protective function during ferroptosis into account, we examined macrophage viability upon RSL-3 treatment. Cells were treated with RSL-3 in the absence or presence of Liproxstatin-1 for 24 h, followed by incubations with CellTiter-Blue for 1 h (Fig. 4C). No changes in viability became apparent, neither under normoxia nor hypoxia. Along these lines, malondialdehyde (MDA) formation, which is generated during decomposition of unstable peroxides derived from polyunsaturated fatty acids, remained unaltered (Fig. 4D). It appears that RSL-3 induces FTH and FTMT in macrophages under normoxia and even stronger under hypoxia but fails to initiate ferroptosis in these cells. This opened the possibility that FTMT and FTH rather protect macrophages from ferroptosis.

### 3.7 FTMT and FTH interfere with ferroptosis under hypoxia

To address a potential ferroptotic protective role of FTMT or FTH in human macrophages, cells were transfected with siRNA against FTMT (siFTMT), FTH (siFTH), both ferritins (siFTMT/FTH), or a control siRNA (NCT) (Fig. S3B). Afterwards they were treated with RSL-3, followed by viability determinations (Fig. 4E). Only with a knockdown of either FTMT or both ferritins RSL-3 was able to reduce cell viability under normoxia, while the siRNA approach to lower FTH showed a trend similar to FTMT but did not reach significance. Knocking down FTMT under hypoxia did not allow RSL-3 to reduce cell viability. However, with a knockdown of both, FTMT and FTH hypoxic cells became sensitive to RSL-3.

To ensure that decreased viability in siFTH/FTMT cells indeed occurs via ferroptosis we intended to rescue macrophage death by using Liproxstatin-1 (Fig. 4F). Under hypoxia the reduced viability in RSL-3-treated siFTMT/FTH cells was reversed by Liproxstatin-1. Reduced cell viability seen with RSL-3 in siFTMT/FTH cells under normoxia remained unaffected by Liproxstatin-1 and thus, likely is a result of cell transfection but unrelated to ferroptosis. These data indicate that under hypoxia both ferritins are protective, which goes in line with the remarkable induction of FTMT and FTH upon RSL-3 treatment (Fig. 4A and B).

**Fig. 5:**
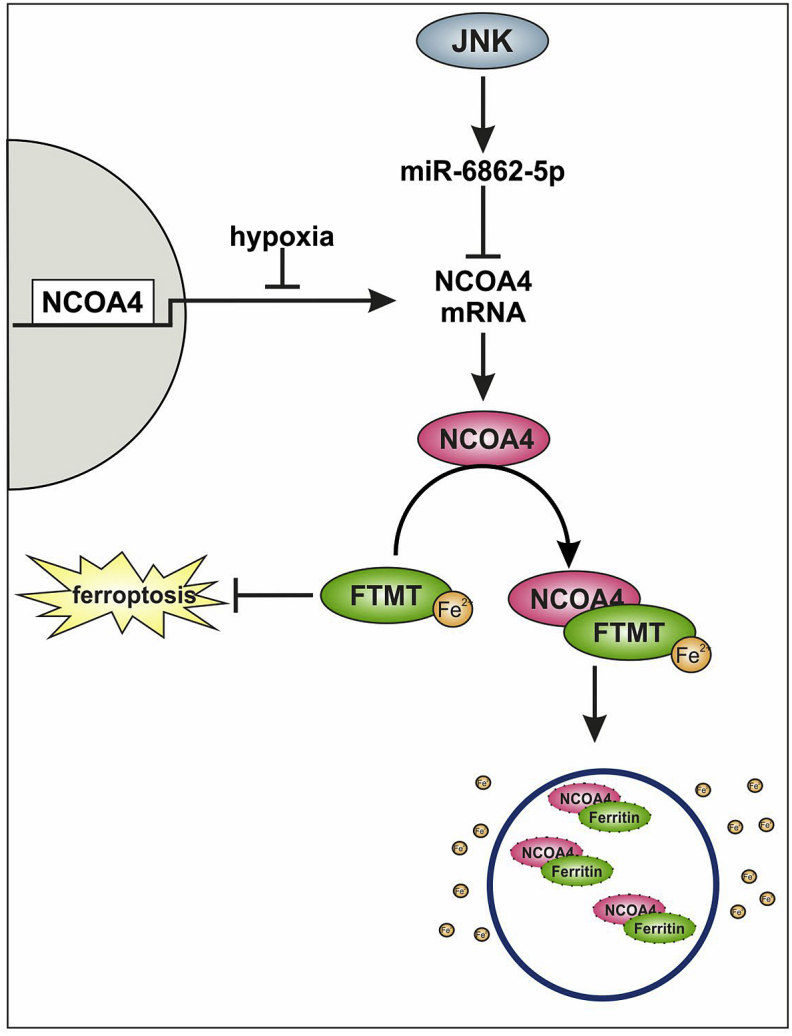
Proposed mechanism of NCOA4 regulation under hypoxia. In human macrophages nuclear receptor coactivator 4 (NCOA4) mRNA is reduced under hypoxia by decreased transcription and in parallel by micro RNA (miR)-6862-5p, which is regulated by c-Jun N-terminal kinase (JNK). As result, NCOA4 protein and consequently ferritinophagy are decreased. In turn, this increases FTMT abundance under hypoxia, which protects from ferroptosis.

In macrophages JNK acts on miR-6862-5p to degrade NCOA4 mRNA. Taking also into consideration that hypoxia decreases transcription of NCOA4 mRNA these combined actions lower protein amount of NCOA4 (Fig. 5). As diminished levels of NCOA4 dampen ferritinophagy ferritins, especially FTMT, increases with the ability to interfere with RSL-3-induced ferroptosis. Since ferroptosis is discussed as a strategy for tumor therapy and hypoxia appears as a cardinal sign of many tumors, we intended to explore the role of ferritins and NCOA4 in ferroptosis of HT1080 fibrosarcoma cells [[42], [43], [44]].

### 3.8 Tumor cells show a high NCOA4 but low FTMT expression profile

**Fig. 6:**
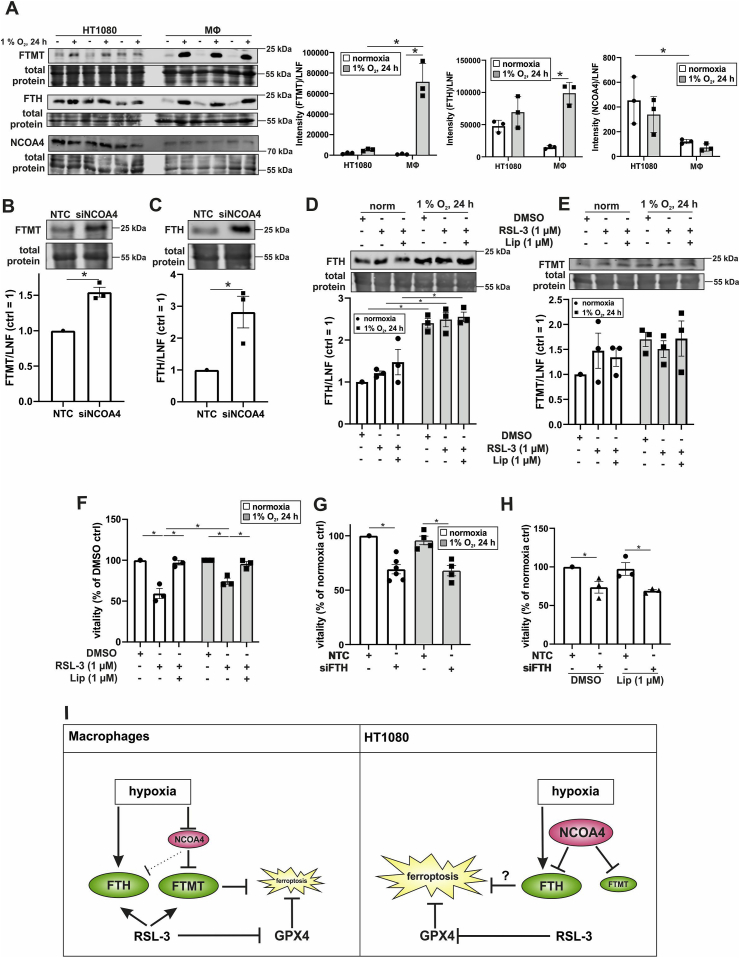
Regulation of ferritinophagy in HT1080 cells. A. Primary human macrophages and HT1080 cells were incubated for 24 h under hypoxia: FTMT, FTH, and NCOA4 were analyzed by Western blotting. For quantification, intensities were normalized to lane normalization factor (LNF) (n = 3). B–C. HT1080 cells were transfected with a siRNA against NCOA4 and Western analysis was performed for FTMT and FTH. Intensities were normalized to LNF (n = 3). D-E. HT1080 cells were incubated for 24 h under hypoxia and treated with RSL-3 (1 μM) and Liproxstatin-1 (Lip, 1 μM) within the last 4 h of incubation. Western analysis was performed for FTH and FTMT (n = 3). F. HT1080 cells were treated as in D and vitality was assessed by CellTiter blue assay (n = 3). G. HT1080 cells were transfected with a siRNA against FTH and incubated for 24 h under hypoxia followed by CellTiter-Blue assays (n = 4–6). H. HT1080 cells were transfected with a siRNA against FTH and treated with Lip directly after transfection. Vitality was measured by the CellTiter-Blue assay (n = 3). I. Proposed mechanism of ferroptosis in human macrophages vs. HT1080 cells. For details see the text. Complete total protein stains are collected in Fig. S1. All data are depicted with SEM. Students t-test or ANOVA values p < 0.05 were considered as significant.

In macrophages a very well controlled system operating via NCOA4 and ferritin expression protects against ferroptosis under hypoxia. As most studies on ferroptotic cell death are done in tumor cells, we explored NCOA4 and ferritin expression in HT1080 cells in direct comparison to macrophages (Fig. 6A). Hypoxia induced FTMT in HT1080 cells, albeit to a lower degree compared to macrophages. Basal expression of FTH was higher in HT1080 cells compared to macrophages but they only responded weakly to hypoxia. Normoxic levels of NCOA4 were higher in HT1080 cells compared to macrophages but they did not downregulate NCOA4 under hypoxia. We then used siRNA to knockdown NCOA4 in HT1080 cells. This provoked an increase of FTMT and FTH, suggesting regulation of both proteins by NCOA4 (Fig. 6B). In contrast to a well-orchestrated ferritinophagy in macrophages, HT1080 cells showed a different expression pattern of NCOA4 and FTH. In HT1080 cells NCOA4 was constitutively expressed, which may indicate a high basal level of ferritinophagy. However, this appears to be compensated by increased FTH levels. In contrast to macrophages, in HT1080 cells neither FTH nor NCOA4 were sensitive to hypoxia. Macrophages decreased NCOA4 and thus, ferritinophagy under hypoxia, thereby increasing FTMT. In the case of HT1080 cells, NCOA4 is not regulated and a constitutive degradation of FTMT can be considered. Consequently, HT1080 cells lose the ability to increase FTMT protein under hypoxia. We finally questioned the role ferritins in tumor cell ferroptosis.

### 3.9 Hypoxia protects tumor cells from RSL-3-induced ferroptosis

In hypoxic macrophages either FTMT or FTH was sufficient to protect from ferroptosis. As HT1080 cells basally express FTH, but only low levels of FTMT, we asked whether FTH protects cells from ferroptosis under hypoxia. Therefore, HT 1080 cells were incubated for 20 h under hypoxia and treated for 4 h with RSL-3 plus/minus Liproxstatin-1. For HT1080 cells already 1 μM RSL-3 was sufficient to induce ferroptosis, whereas 10 μM was needed in macrophages. Morphologically, RSL-3-treated cells lose their fibroblastic shape and get roundish, which was suppressed by Liproxstatin-1. The RSL-3-induced morphology changes appeared also to be reduced under hypoxia (data not shown). In hypoxic HT1080 cells the expression of FTH, but not FTMT significantly increased and was not affected by RSL-3 or Liproxstatin-1 (Fig. 6D and E).

Checking cell viability with CellTiter-Blue showed no significant differences, when comparing normoxic to hypoxic HT1080 cells (Fig. 6F, S4A). RSL-3 lowered viability of HT1080 by roughly 50%, which was fully compensated by Liproxstatin-1. Under hypoxia RSL-3-treatment was less efficient, reducing cell viability by only about 25%, which was again due to ferroptosis. To molecularly define the role of FTH in HT1080 cell death, cells were treated with a siRNA against FTH and incubated for 24 h under hypoxia (Fig. 6G). Interestingly, already the knockdown of FTH decreased cell viability under normoxia, which was not further decreased by the addition of RSL-3 (Fig. S4B). Similar observations were made under hypoxic conditions. Apparently, FTH is essential for HT1080 cell survival. This raises the possibility that a knockdown of FTH may increase free iron, initiate Fenton chemistry-associated lipid peroxidation, and ferroptosis. To explore whether death of HT1080 cell as a result of the FTH knockdown is a ferroptotic one, we tested Liproxstatin-1 (Fig. 6H). As Liproxstatin-1 failed to rescue from cell demise under these conditions cell death is not via ferroptosis. However, as hypoxia partially protected HT1080 cells from ferroptosis and it still remains an open question whether this is due to an increase in FTH or a lack of oxygen and consequently, reduced lipid peroxidation.

Induction of ferroptosis by pharmacological interference either by inhibition of the X^-^ -transporter or GPX4 is an emerging concept for cancer therapy [43,44]. For stromal cells, i.e. macrophages and cancer cells our data suggest, that hypoxic cells are more resistant to RSL-3 than normoxic cells, which might suggest that hypoxic tumor regions are less vulnerable. Targeting FTH may turn out as the Achilles heel of cancer cells and an interesting candidate for further studies.

The emerging picture suggests that in macrophages hypoxia decreases NCOA4 and thereby increases the levels of FTMT and FTH (Fig. 6I). Both proteins are, by so far unknown reasons, further enhanced by RSL-3 and protect from ferroptosis. In HT1080 tumor cells NCOA4 is unresponsive to a decrease in oxygen and therefore expression of FTMT remains largely unaffected by hypoxia, while FTH increases. Based on our studies we can discriminate between a hypoxic and a RSL-3-mediated induction of FTH and FTMT. The inverse correlation between NCOA4 and FTMT in hypoxic macrophages was not seen in HT1080 cells. In contrast, expression of FTH, which appeared to be NCOA4-independent under hypoxia, increased in macrophages and HT1080 cells. One can speculate that the increase of FTH under hypoxia protects HT1080 cells from ferroptosis. The increase of FTH and FTMT by RSL-3, which was macrophage specific, may add to protect these cells in addition to hypoxic regulation of these players. Apparently, FTMT, FTH, and their regulation by NCOA4 appear important to modulate sensitivity of myeloid cells to ferroptosis. Further studies are needed to mechanistically understand how RSL-3 induces FTH and FTMT. It will also be necessary to clarify whether results seen in HT 1080 can be generalized for other fibrosarcoma and to elucidate a potential combinatorial approach of inducing ferroptosis and targeting ferritinophagy.

## Funding

This work was supported by the 10.13039/501100001659Deutsche Forschungsgemeinschaft (DFG), Germany [SFB815, project A8 (B.B)].

We declare no conflict of interest.

## Declaration of competing interest

All authors concur with this submission. Data have neither been submitted previously nor are under consideration for publication elsewhere. We declare no conflict of interest.
