# Supplementary material for: Hypoxia inhibits ferritinophagy, increases mitochondrial ferritin, and protects from ferroptosis
Source: Redox Biol. 2020 Aug 3;36:101670. doi: 10.1016/j.redox.2020.101670 (PMC7452134; doi:10.1016/j.redox.2020.101670)
Supplement: Multimedia component 1 [file mmc1.pdf]

Figure 2

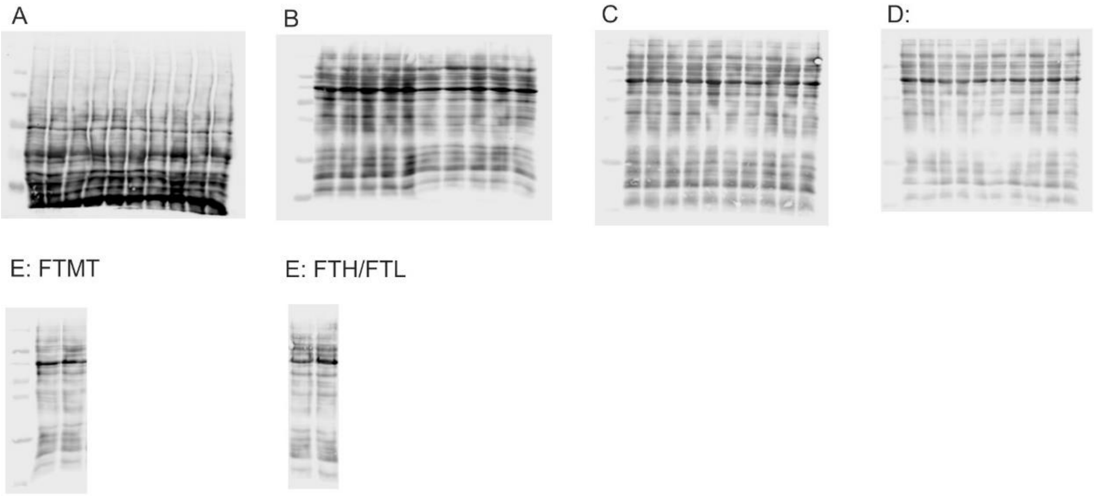

Figure 3

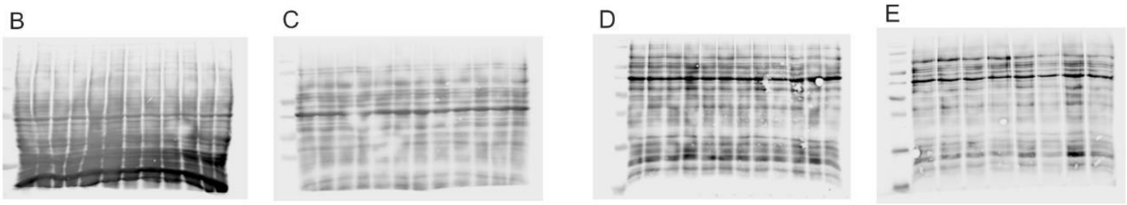

Figure 4

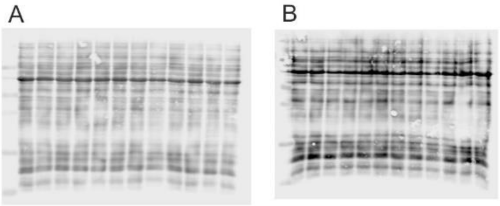

Figure 6

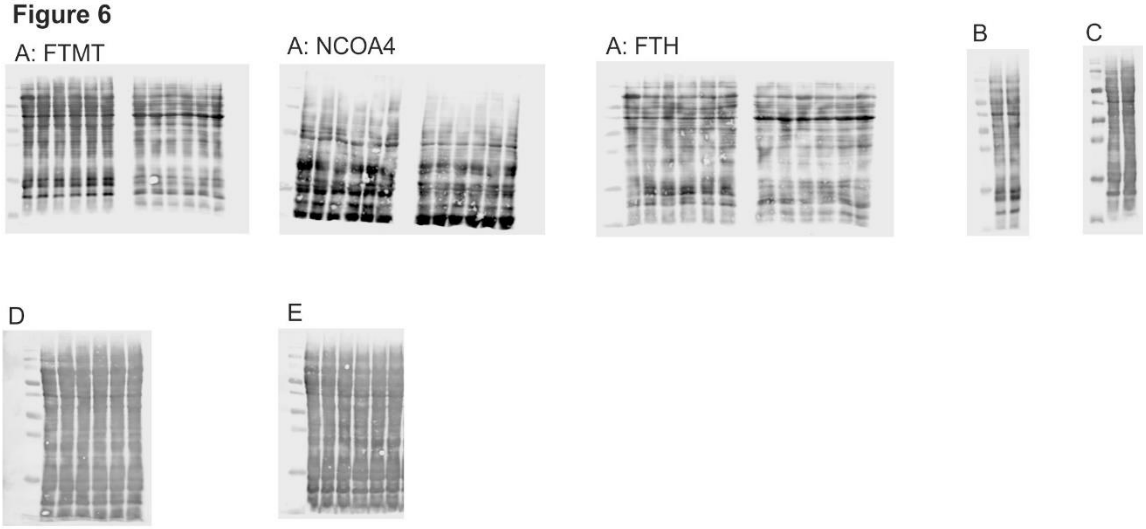

Figure S1: Total protein stain

Membranes were stained with Revert 700 Total Protein Stain kit before blocking. The figure shows the complete membrane of every blot, which was used to create the figures within this publication.

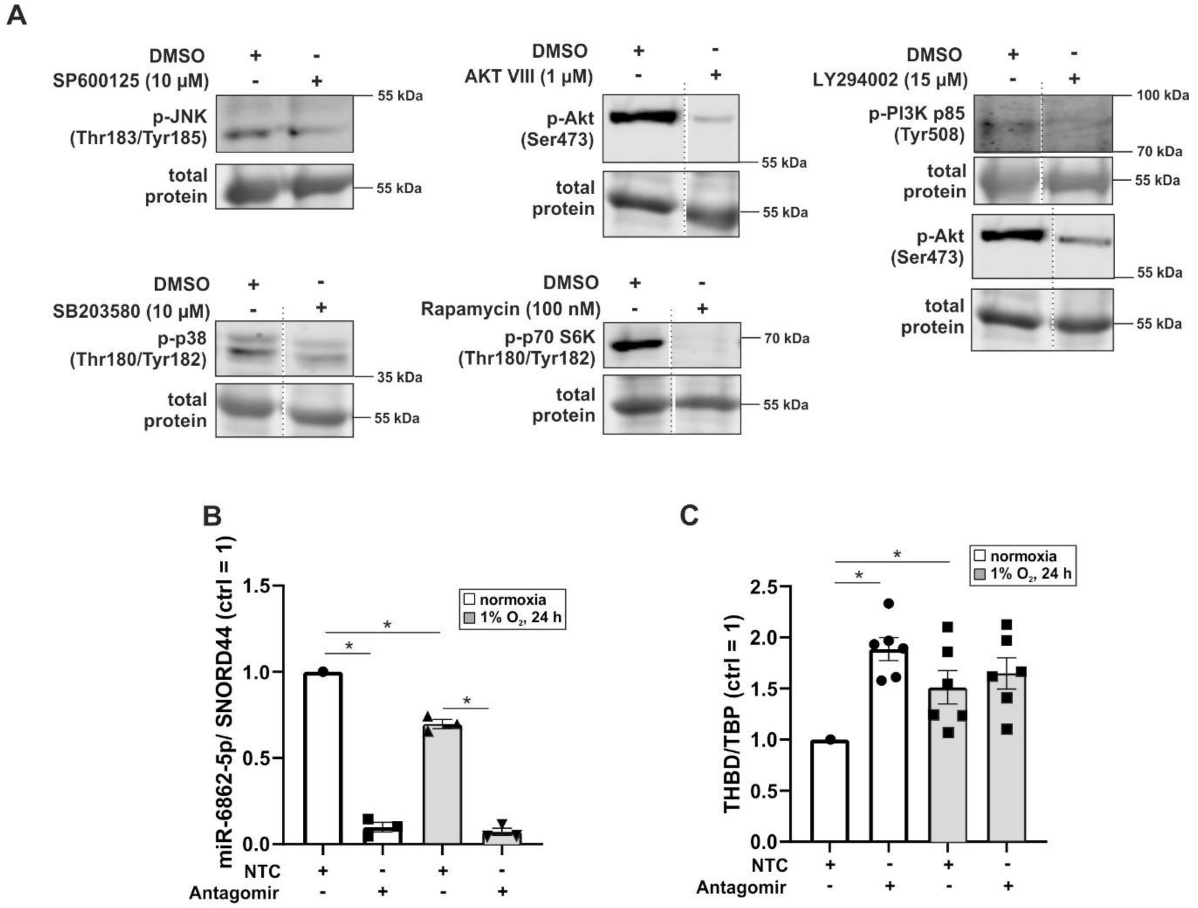

Figure S2: Validation experiments: Kinase inhibitors and the miR-6862-5p knockdown

A. Primary human macrophages were treated with kinase inhibitors at indicated concentrations for 24 h, followed by Western analysis for phosphorylated (p-) c-Jun N-terminal kinase (JNK), protein kinase B (Akt), phosphoinositide 3-kinase p85α (PI3K p85), p38 mitogen-activated protein kinase (p38), and p70 S6 kinase (p70S6K). Each individual gel was loaded with the DMSO control and inhibitor-treated samples (DMSO, SP600125, AKT VIII, LY294002, SB203580 or rapamycin). After blotting, the membranes were stained against the different kinases. Each figure shows phosphorylation of a distinct kinase in the DMSO control vs. the kinase-blocked sample, i.e. p-AKT vs p-AKT in the presence of AKT VIII. For clarity, the band showing the impact of SP600125 in the p-Akt blot was cut out (indicted by the dashed line). Thus, showing controls and inhibited kinases are from the same blot and samples treated with Akt VIII- and LY294002 in the p-Akt blot refer to the same control. B and C. Primary human macrophages were transfected with a non-targeting control (NTC) or a miR-6862-5p antagomir. B. Analysis of miR-6862-5p expression under normoxia and hypoxia. Expression was normalized to SNORD44. Normoxic NTC was set to 1 (n = 3). C. Thrombomodulin (THBD) expression was analyzed by qPCR and normalized to TBP. Normoxic NTC was set to 1 (n = 6). All data are expressed as SEM. Students t-test values p <0.05 were considered as significant.

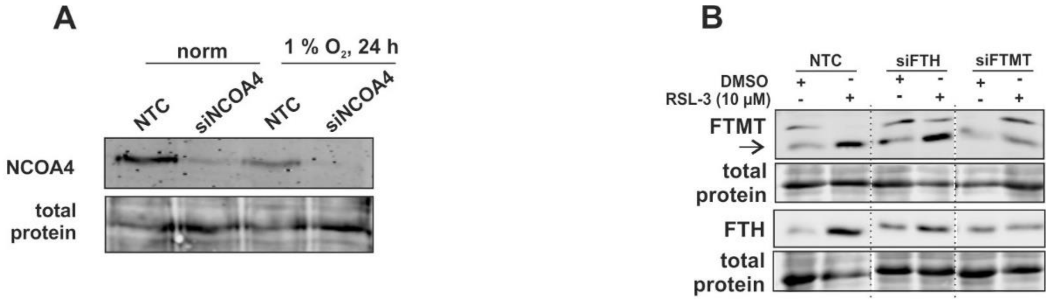

Figure S3: Validation of siRNA-mediated knockdowns

A. Primary human macrophages were transfected with siRNA against NCOA4 (siNCOA4) or a non-targeting control (NTC), incubated under hypoxia, and expression of the protein was analyzed by Western blot. B. Human macrophages were transfected with siRNA against ferritin heavy chain (siFTH) or mitochondrial ferritin (siFTMT) and compared to a non-targeting control (NTC). To validate the functionality of the siRNA, FTH and FTMT were induced by RSL-3 (10 µM). Expression of FTH and FTMT was determined by Western analysis. For reasons of clarity the blot was cut at the dashed lines.

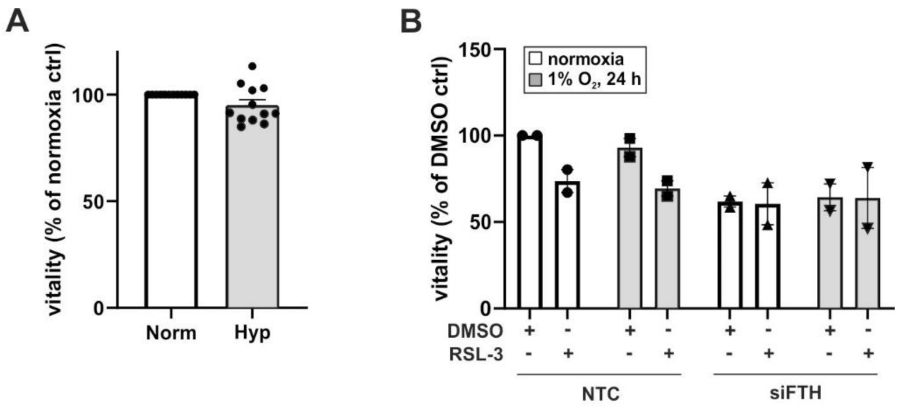

Figure S4: Vitality of HT1080 cells

A. Vitality of HT1080 cells was measured by CellTiter-Blue assay under normoxia and after hypoxic incubation (1% O_2, 24 h) (n = 12). B. HT1080 cells were transfected with a siRNA against ferritin heavy chain (FTH) or a non-targeting control (NTC) and incubated for 24 h under hypoxia. For the last 4 h cells were treated with RSL-3 (1 μM) (n = 2).
